# Network-Based Prediction of Novel CRISPR-Associated Genes in Metagenomes

**DOI:** 10.1128/mSystems.00752-19

**Published:** 2020-01-14

**Authors:** Jake L. Weissman, Philip L. F. Johnson

**Affiliations:** a Department of Biology, University of Maryland, College Park, Maryland, USA; Mayo Clinic

**Keywords:** CRISPR, metagenomics, microbial ecology, network

## Abstract

A diversity of clustered regularly interspaced short palindromic repeat (CRISPR)-Cas systems provide adaptive immunity to bacteria and archaea through recording “memories” of past viral infections. Recently, many novel CRISPR-associated proteins have been discovered via computational studies, but those studies relied on biased and incomplete databases of assembled genomes. We avoided these biases and applied a network theory approach to search for novel CRISPR-associated genes by leveraging subtle ecological cooccurrence patterns identified from environmental metagenomes. We validated our method using existing annotations and discovered 32 novel CRISPR-associated gene families. These genes span a range of putative functions, with many potentially regulating the response to infection.

**IMPORTANCE** Every branch on the tree of life, including microbial life, faces the threat of viral pathogens. Over the course of billions of years of coevolution, prokaryotes have evolved a great diversity of strategies to defend against viral infections. One of these is the CRISPR adaptive immune system, which allows microbes to “remember” past infections in order to better fight them in the future. There has been much interest among molecular biologists in CRISPR immunity because this system can be repurposed as a tool for precise genome editing. Recently, a number of comparative genomics approaches have been used to detect novel CRISPR-associated genes in databases of genomes with great success, potentially leading to the development of new genome-editing tools. Here, we developed novel methods to search for these distinct classes of genes directly in environmental samples (“metagenomes”), thus capturing a more complete picture of the natural diversity of CRISPR-associated genes.

## Abstract (precis)

Every branch on the tree of life, including microbial life, faces the threat of viral pathogens. Over the course of billions of years of coevolution, prokaryotes have evolved a great diversity of strategies to defend against viral infections. One of these is the CRISPR adaptive immune system, which allows microbes to “remember” past infections in order to better fight them in the future. There has been much interest among molecular biologists in CRISPR immunity because this system can be repurposed as a tool for precise genome editing. Recently, a number of comparative genomics approaches have been used to detect novel CRISPR-associated genes in databases of genomes with great success, potentially leading to the development of new genome-editing tools. Here, we developed novel methods to search for these distinct classes of genes directly in environmental samples (“metagenomes”), thus capturing a more complete picture of the natural diversity of CRISPR-associated genes.

## OBSERVATION

Every branch on the tree of life, including microbial life, faces the threat of viral pathogens. Over billions of years of coevolution with their viruses, prokaryotes have developed numerous strategies for defending themselves (1). The study of these defense systems has drawn much attention of late, not only for their ecological implications but also for their ability to target specific DNA or RNA sequences, making them powerful tools for handling and editing genetic material. The clustered regularly interspaced short palindromic repeat (CRISPR) immune system, widespread in both bacteria and archaea, enables us to designate specific sites that we would like to edit on a genome and either to corrupt or to add to the sequence in those locations (2). CRISPR systems are diverse, with system types and subtypes distinguished by distinct sets of associated protein machinery (3). This diversity has sparked something of a gold rush to characterize novel CRISPR proteins and other microbial defense systems. Just since 2018, dozens of novel CRISPR-associated (*cas*) genes as well as more than 10 entirely novel classes of defense systems have been discovered (4–6).

Several groups have discovered large numbers of putative *cas* genes by exploiting the fact that *cas* genes are often colocated on the genome and looking for gene families which frequently show up adjacent to known *cas* genes (4, 5). This approach has led to the discovery of numerous novel proteins that may be involved in the mechanism or regulation of CRISPR immunity. Nevertheless, such studies have relied on publicly available databases of assembled genomes, which capture only a small and strongly biased subset of the total range of global microbial diversity. In order to paint a more complete picture of functional CRISPR diversity, we mined a large data set of ocean metagenomes (7) for novel *cas* genes. In place of physical proximity on the genome, our approach instead exploits ecological relationships—detectable via gene cooccurrence patterns.

Recently, large-scale metagenomic data sets have permitted the construction of functional networks connecting groups of orthologous genes on the basis of how strongly their abundances correlate across independent samples (8). These networks can be used for gene annotation, taking advantage of predictive techniques developed extensively in coexpression and protein-protein interaction networks (see, e.g., reference 9). Such an approach works especially well for defense genes, where frequent horizontal transfer and loss events should reduce spurious correlations due to changes in the taxonomic composition of a community (10).

**FIG 1:**
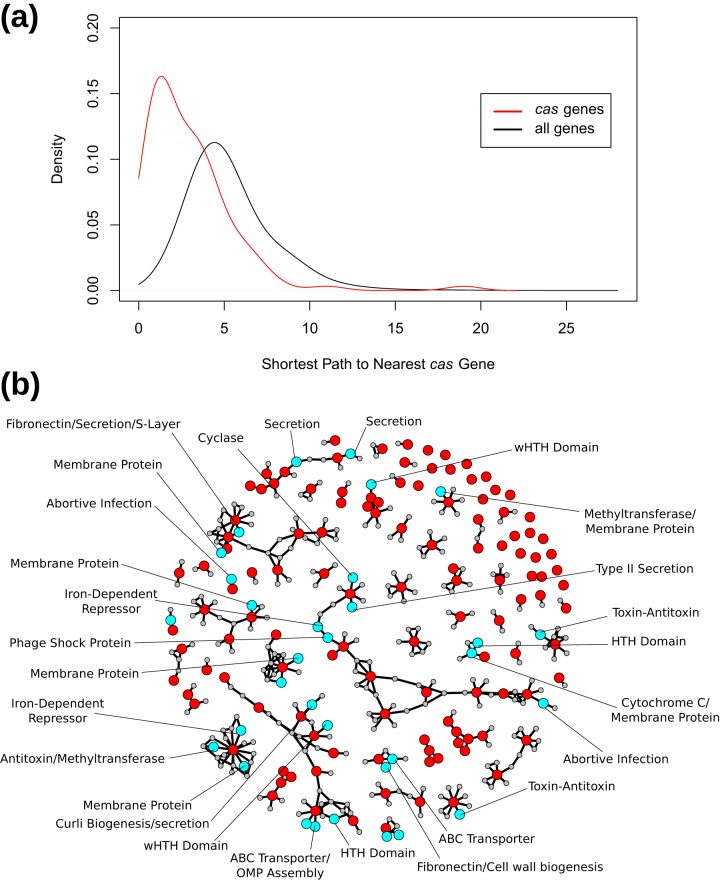
Prediction and annotation of novel CRISPR-associated (*cas*) gene families from a globally distributed set of metagenomes. (a) Known CRISPR-associated genes cluster close to each other in the network. This result validates the assumption of label propagation methods that adjacent nodes in the network have similar features. (b) Predicted *cas* genes (cyan), known *cas* genes (red), and their neighbors (gray) shown in the network. The full network is not shown due to its size. Sensitive profile-profile searches suggested functional annotations for many of these genes (shown as labels). wHTH, winged helix-turn-helix domain.

We built a conditional-dependence network from abundance profiles of eggNOG (11) gene families (“NOGs”) provided by the Tara Oceans project (7) using an approximation to the graphical lasso (12) combined with tools for large-scale network construction (13). The resulting network revealed that *cas* gene families cluster more closely to other *cas* gene families than to genes chosen at random (Fig. 1a), indicating that proximity on the network can be used to predict gene function. Proximity-based label propagation methods rely on this “guilt-by-association” assumption, spreading annotations across the network between closely connected nodes. We predicted 32 novel putative CRISPR-associated gene families using a neural-network-based label propagation method specialized for annotation problems with a small number of positive annotations (Fig. 1b) (9). Given that the network consists of 28,988 nodes, 9,842 of which have a preexisting annotation and only 122 of which are *cas*, our prediction problem is one of finding a needle in a very large haystack. Nevertheless, 5-fold cross-validation with known *cas* genes showed this method to be conservative but effective (see Fig. S1 in the supplemental material), with a low false-positive rate (7 × 10^−4^), and that it was able to successfully recover about one in six *cas* gene families (true-positive rate of 0.16; successfully predicted genes had highly correlated abundances corresponding to other known *cas* genes across samples) (Fig. S2). We emphasize that these gene families are “CRISPR-associated” families in a somewhat loose sense, in that many are likely involved in regulating the response to viral infection (see below) rather than being directly implicated in the targeting of viral sequences or acquisition of novel immune memories. For simplicity, we refer to our predicted genes as *cas* genes here, though these gene families may appear by themselves and/or provide nonimmune functions in other contexts (though the same can be said even of the universal *cas* gene *cas1* [14]). When inferring function in a specific genome, then, it is likely that one would have to consider more than simple homology (e.g., genomic context, transcriptional patterns).

10.1128/mSystems.00752-19.1FIG S1Performance of COSNet prediction on the network under 5-fold cross validation. The regularized and nonregularized versions of the model had similar performance. For comparison, we also included a null model that included only the total number of *cas* genes in the network and predicted their locations at random. The F1 score is defined as $2\times \left(\frac{\text{precision}\times \text{recall}}{\text{precision}+\text{recall}}\right)$. Download FIG S1, PDF file, 0.02 MB.Copyright © 2020 Weissman and Johnson.2020Weissman and JohnsonThis content is distributed under the terms of the Creative Commons Attribution 4.0 International license.

10.1128/mSystems.00752-19.2 FIG S2 Correlated abundances enable accurate prediction of *cas* proteins. (a) Correlation matrix of abundances of the 122 known *cas* proteins (normalized by single-copy marker genes; see the methods described in the text) across samples. The bar on the left indicates whether this gene family was accurately predicted to be *cas* by label propagation during cross validation (blue) or was missed (red). Note that accurately predicted gene families are often members of tightly correlated clusters of genes. (b) Correctly predicted *cas* protein families tended to be highly correlated with at least one other *cas* gene to a greater degree than the families that were missed by the algorithm. (c) In general, looking at all pairwise correlations between the 122 families, known *cas* genes tend to have correlated abundances (*t* test, *df *= 7,380, *P < *2.2 × 10^−16^). The mean value (0.17) is denoted with a dashed line. Download FIG S2, PDF file, 0.1 MB. Copyright © 2020 Weissman and Johnson. 2020 Weissman and Johnson This content is distributed under the terms of the Creative Commons Attribution 4.0 International license.

Most of the 32 gene families that we predicted to be *cas* would not have been detected by the proximity-based methods previously employed by others. Searching among all completely assembled genomes on GenBank, we found none of the 32 families with a signal of proximity to *cas* genes (Fig. S3). This result is to be expected, given that the distribution of strains and defense genes in the ocean is likely poorly represented by public databases of sequenced genomes. Therefore, we repeated our search using a set of 2,631 metagenome-assembled genomes from the ocean (15). Only 5 of our predicted families were significantly more likely than random to appear on the same contig as a known *cas* gene in these genomes (for NOG85832, *P < *10^−4^; for NOG121080, *P = *0.0021; for NOG121689, *P < *10^−4^; for NOG269516, *P < *10^−4^; for NOG273942, *P = *10^−4^). However, this lack of proximity may reflect a lack of power (due to an inability to assemble much of the original metagenomes) rather than a true absence of proximity.

10.1128/mSystems.00752-19.3 FIG S3 Putative novel *cas* genes were not found specifically near known *cas* genes in assembled genomes from a public database. Histograms show minimum distances between putative and known *cas* genes across genomes in GenBank. Black lines show null distributions of distances to *cas* with randomly drawn sets of genes. Putative *cas* NOG10439 is not shown as it was not found in this set of genomes. Download FIG S3, PDF file, 0.7 MB. Copyright © 2020 Weissman and Johnson. 2020 Weissman and Johnson This content is distributed under the terms of the Creative Commons Attribution 4.0 International license.

We found that 5 genes in our putative novel *cas* families (NOG87308, NOG121080, NOG145673, NOG273942, and NOG314802) were homologous to known *cas* genes in comparisons to a large protein database (see Table S1 in the supplemental material for details; hmmsearch E value cutoff 0.05). Notably, NOG12180 and NOG273942 showed both signatures of homology and proximity to known *cas* genes, in addition to our ecology-based prediction, making them strong candidates for future research.

10.1128/mSystems.00752-19.6 TABLE S1 NOGs for our 32 putative new *cas* genes with homology to proteins in UniProt containing the keyword “CRISPR.” All hits are included for NOGs containing at least one match with an E value of <0.05. Download Table S1, PDF file, 0.04 MB. Copyright © 2020 Weissman and Johnson. 2020 Weissman and Johnson This content is distributed under the terms of the Creative Commons Attribution 4.0 International license.

**FIG 2:**
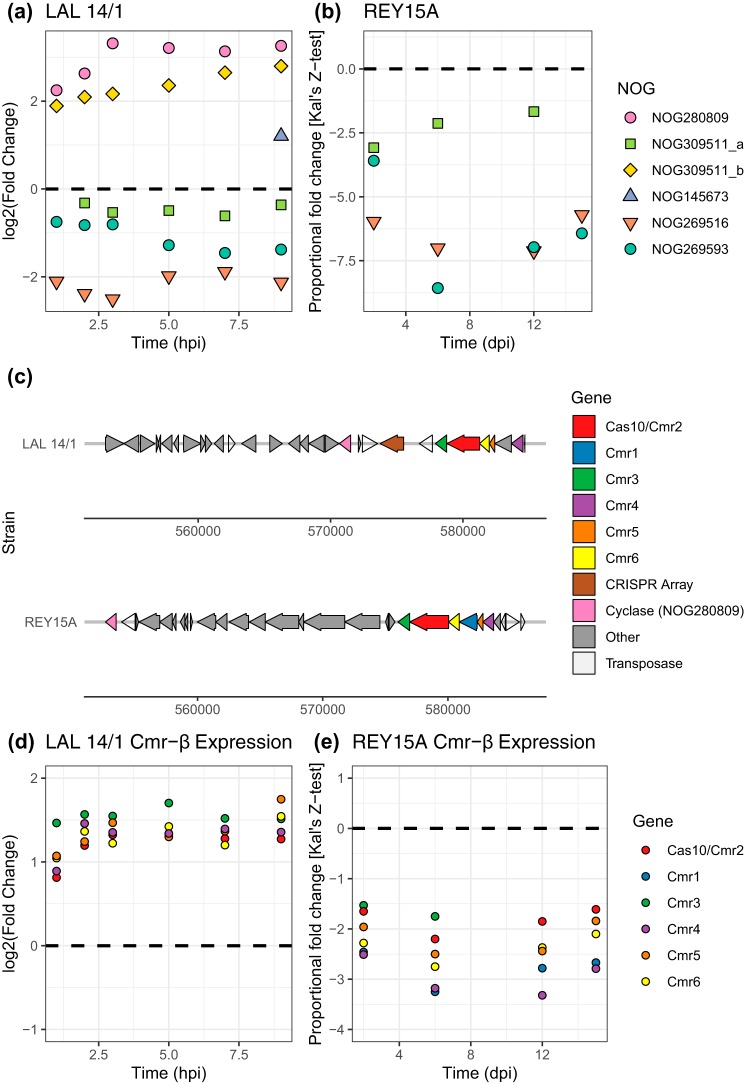
Differential expression of putative novel *cas* genes in Sulfolobus islandicus strains LAL14/1 and REY15A during infection (16, 17). (a and b) Of the 6 novel *cas* genes harbored in these two genomes (a), all 6 were significantly differentially expressed in strain LAL14/1, whereas (b) only 3 were differentially expressed in REY15A. (c) NOG280809 is located nearby the *cas* operon Cmr-β in LAL14/1, where it is upregulated, but is much farther away in REY15A, where it is not differentially expressed. Notably, this region contains a number of transposases and appears to have undergone considerable rearrangement. (d and e) The *cas* operon Cmr-β is upregulated during infection in LAL14/1 (d) but downregulated in REY15A (e). To aid in the comparisons, dashed horizontal lines denote the control uninfected expression level in panels a and b and panels d and e. Points above or below these lines denote upregulation or downregulation, respectively. Multiple instances of gene families are distinguished using letter suffixes (e.g., “_a”). Note that in addition to the use of different host strains, the two data sets use different viruses, measure and analyze expression data differently, and examine different time scales of infection (hours postinfection [hpi] versus days postinfection [dpi]) (16, 17).

To determine if we could directly implicate any of these genes in the host’s response to infection, we analyzed two data sets detailing the transcriptional response of different Sulfolobus islandicus strains (LAL14/1 and REY15A) to viral infection (16, 17). We found representatives of 6 of our predicted families in the S. islandicus genomes, all of which were differentially expressed in LAL14/1 but only 3 of which were differentially expressed in REY15A (Fig. 2a and b). In particular, an ortholog of NOG280809, annotated as a cyclase, was strongly upregulated at all postinfection time points for LAL14/1 and was located three genes upstream of a CRISPR array (2,166 bp), near the Cmr-β type III-B *cas* operon (Fig. 2c). Interestingly, in REY15A, a local genomic rearrangement has led to the NOG280809 ortholog being located much further away from the *cas* operon, and it was no longer differentially expressed during infection (despite this gene having 99.9% sequence similarity at the nucleotide level between the two strains). Perhaps related to this rearrangement, the Cmr-β operon was upregulated during infection in LAL14/1 but was downregulated in REY15A, warranting further experimental investigation (Fig. 2d and e). That said, the differences in experimental procedures between the two studies make further conclusions difficult (see below) (16, 17). Additional analysis of an expression data set from the bacterium Thermus thermophilus also revealed that many of the putative novel *cas* genes in this organism’s genome are differentially expressed during infection (Fig. S4 and S5) (18).

10.1128/mSystems.00752-19.4 FIG S4 Expression of putative novel *cas* genes in Thermus thermophilus during infection (18). One putative novel *cas* gene (an ortholog of NOG315893) was found in the genome but was not differentially expressed under any condition and is not shown. (a) Wild-type expression postinfection. (b) Expression in a Δ*crp* strain, which was observed to show reduced postinfection expression of the known *cas* targeting genes but increased expression of the acquisition machinery (Fig. S5) (18). (c) With the exception of NOG315892_d, all putative novel *cas* genes were found on the host chromosome, whereas all known *cas* operons are located on a single plasmid (18). NOG315892_d is found about 5 kb upstream of a CRISPR array on the plasmid. Multiple instances of a gene family are distinguished using letter suffixes (e.g., “_a”). Download FIG S4, PDF file, 0.02 MB. Copyright © 2020 Weissman and Johnson. 2020 Weissman and Johnson This content is distributed under the terms of the Creative Commons Attribution 4.0 International license.

10.1128/mSystems.00752-19.5 FIG S5 Expression of known *cas* operons in Thermus thermophilus during infection (18). All operons found on a single plasmid, with none found on the host chromosome. The Δ*crp* strain showed reduced postinfection expression of the *cas* targeting genes relative to the wild-type strain (b and h) but increased expression of the acquisition machinery (18). Download FIG S5, PDF file, 0.02 MB. Copyright © 2020 Weissman and Johnson. 2020 Weissman and Johnson This content is distributed under the terms of the Creative Commons Attribution 4.0 International license.

To functionally characterize our set of putative *cas* genes, we applied profile-profile searches to reveal distant homologies to known gene families based on protein structure (19). Several putative *cas* genes were annotated as components of abortive infection (Abi) or toxin-antitoxin type defense systems, which kill the cell upon infection to prevent further spreading of the virus to nearby kin. Other groups have suggested that Abi and DNA-degrading systems such as CRISPR often come as paired defense strategies (20). Alternatively, CRISPR systems are often inducible in response to infection (see, e.g., reference 21). Such inducibility requires recognition of viral infection, similar to what would be needed for an Abi-type defense. It is plausible that some CRISPR systems have co-opted the response capabilities of Abi systems to detect viruses, consistent with the observation that CRISPR systems often evolve by swapping out and in parts of their machinery in a modular fashion (3).

Several other putative *cas* genes had potential infection-sensing function (Table 1). Notably, we annotated one family as representing a putative phage shock protein, typically responding to changes in membrane permeability. Another family contains cyclases, which are potentially relevant as type III CRISPR systems are thought to use cyclic molecules to coordinate their response to infection (22). A number of families were predicted to contain DNA-binding domains common in *cas* genes. Four families were annotated as having a helix-turn-helix (HTH) DNA-binding domain, with two of these being the winged HTH domain which is common in *cas* genes (23). Two additional genes were predicted to be possible iron-dependent repressors, typically containing a winged helix domain at their N terminus (24).

**TABLE 1:** Putative novel *cas* genes and their annotations

NOG	Putative type	Regulatory role	Defense role^*c*^	Membrane or extracellular role	TM^*d*^
NOG10439				ABC transporter	X
NOG16349				Major facilitator superfamily	X
NOG44531		PspC			X
NOG46784			TA		
NOG82932	I-U				
NOG84780			TA		
NOG85832^*a*^				Type II secretion, peptidoglycan binding	X
NOG87308^*a*^	I-E^*b*^		Abi		
NOG116663	III-A	HTH domain			
NOG121080^*a*^				Fibronectin, cell wall biogenesis	X
NOG121689^*a*^	I-D	Winged helix			
NOG121881	I-B				
NOG131471	III-A			ABC transporter, outer membrane protein assembly	X
NOG133718		Iron-dependent repressor			
NOG138333	I-E^*b*^			Secretion	
NOG140114		Iron-dependent repressor			
NOG145673^*a*^	I-F^*b*^	Winged helix			
NOG146536				Membrane protein	X
NOG242488	I-D			Curli biogenesis/secretion	
NOG269516^*a*^			TA/RM		
NOG269593^*a*^	I-B^*b*^			Fibronectin, secretion, S-layer, sugar binding	
NOG273942^*a*^	III-A				
NOG280809^*a*^		Cyclase			
NOG296050					
NOG300351				Membrane protein	
NOG309511^*a*^	I-E			Accessory Sec system GspB transporter	
NOG309759	I-E				X
NOG312939	I-U				X
NOG314802^*a*^	I		Abi/RM		
NOG315893^*a*^	I-E	HTH domain			
NOG318199^*a*^	I-B				
NOG328008	I-B^*b*^				X

a Validated by at least one independent source of information.

b Confirmed by multiple neighbors in network.

c TA, toxin-antitoxin; RM, restriction modification.

d Transmembrane.

Finally, we annotated almost half (15) of our predicted gene families as involved in secretion, transport, or extracellular matrix assembly or as being transmembrane related (Table 1). Viruses must interact directly with the cell surface in order to successfully infect a cell, and it is likely any infection-sensing capacity in the cell would start at this interface. This result is in agreement with previous proximity-based searches for novel *cas* proteins, which revealed a large number of integral membrane proteins associated with CRISPR (4).

In summary, we validated a novel network-based approach for the automated discovery of *cas* genes directly from metagenomes that exploits the information encoded in ecological relationships between genes and revealed 32 putative novel gene families associated with CRISPR immunity. Using a rigorous cross-validation framework to assess predictive ability, we found our approach to be conservative, making very few false-positive predictions. Thirteen of our predicted families were partially validated using independent sources of information, including homology and proximity to known *cas* genes as well as transcription postinfection (Fig. 1b and 2a and b; see also Fig. S4a and b) (Table 1). Three of these (NOG12180, NOG273942, and NOG280809) are particularly promising candidates for additional study, based on multiple intersecting lines of evidence. Distant homology suggests that most of our 32 predicted gene families are plausibly involved in the cellular response to phage infection, but full characterization of the role of these proteins in antiviral defense will require experimental manipulation. Finally, while genomic colocation has been a fruitful signal of shared function in the past, it is impossible to determine how often genes interact with CRISPR in *trans* without developing proximity-independent methods to infer functional interactions.

## 

### Network construction.

Functional networks connect genes on the basis of shared or interacting functional roles. These interactions are often inferred on the basis of protein-protein interactions or coexpression or, more recently, on the basis of correlated abundances (8). When we say that our approach exploits ecological relationships between genes, we mean that genes that are highly abundant in similar environments (and less abundant in similar environments) are likely to have related functions. By looking at the correlated abundances of genes across environments, we can capture these functional relationships.

We obtained publicly available functional profiles describing the relative abundances of 63,771 eggNOG v3.0 (http://eggnog.embl.de/version_3.0/) (11) gene families across 139 prokaryote-enriched Tara Oceans samples (7). We then normalized the data to get the average copy number per genome in a sample for each gene family (using the median abundance of a curated set of 77 single-copy marker genes) (25). This normalization step allows us to avoid the statistical issues that arise when working with compositional data. Many of the gene families in the data set were exceedingly rare, and we eliminated any families present in less than 10% of samples, leaving 29,988 families. We then built an unweighted conditional-dependence network connecting these families on the basis of their correlated abundances using the pipeline described in the R package “huge” (13), choosing options that allowed construction of such a large network (26). Specifically, we first transformed our data using nonparanormal transformation [function huge.npn()], followed by network inference using the Meinshausen-Bühlmann method (12) over a range of regularization intensities preceded by sure independence screening (26) [options nlambda = 30 and scr = T and method = “mb” in the huge() function]. We selected the optimal regularization parameter using the rotation information criterion [function huge.select()]. This approach yielded a sparse functional network with 44,236 edges.

Many NOGs come with annotations at the protein family level. Additionally, even when a NOG lacks a family-level annotation, the individual genes within that NOG may be annotated. We sought to maximize the number of nodes in the network annotated as *cas* by considering any NOG containing a *cas* gene to represent a CRISPR-related gene family. Using the uniparc database (27), we matched the set of gene identifiers (IDs) associated with each NOG to protein names. If any protein name contained the keyword “CRISPR,” that protein was considered to be encoded by a *cas* gene, and so was the NOG that contained it. In total, we annotated 122 nodes in this network as *cas* genes.

### Prediction on the network.

Label propagation methods represent a broad class of methods used to classify nodes on a network. Typically, these methods start with a set of labeled nodes and iteratively “propagate” these labels to nearby unlabeled nodes, eventually labeling all nodes on the network. Many variants of label propagation methods have been developed, but they all rely on the central assumption that nodes that are close together on the network are more likely to have similar labels. We began by testing this assumption on our network with respect to *cas* genes (our binary labels being *cas* and non-*cas*). We found the shortest paths from all nodes in our network to the nearest annotated *cas* node using the distances() function in the igraph R package (28). As noted above, this revealed that *cas* genes tend to cluster in the network, making label propagation-based classification methods potentially useful tools for *cas* gene prediction.

For prediction of novel *cas* genes, we used the COSNet label-propogation method, implemented in R, specifically designed for semisupervised classification in networks with extremely imbalanced classes (9). First, we assessed performance using 5-fold cross validation, choosing balanced folds and using the find.division.strat() function to ensure roughly equal numbers of *cas* genes in all folds (which were otherwise randomly selected). We calculated performance metrics considering only annotated NOGs (i.e., those with preexisting annotations as *cas* or with any existing NOG-level annotation; NOGs lacking any annotation were included in the prediction step but not in calculating the resulting performances, since “true” positive or negative values could not be assigned). We then predicted our final 32 putative novel *cas* genes using the complete network. For both cases, following the recommendation in the COSNet documentation, we added a small amount (10^−4^) of regularization, though this had little effect on our results from comparisons to a model without regularization (Fig. S1).

We note that our method is biased toward the discovery of class 1 and, in particular, type I systems (Table 1), as these make up the majority of our training set of gene families.

### Proximity-based search.

We downloaded a set of 2,631 draft metagenome-assembled genomes that were assembled from the Tara data set (15). Using hmmer (29), we built profile hidden Markov models (HMMs) of all NOGs annotated as *cas* at the NOG level as well as our set of 32 putative novel *cas* NOGs and searched the complete set of profiles against each genome using hmmscan (E value cutoff of 0.01/number of genomes). We then quantified how often (on average) each of our 32 gene families cooccurred on the same contig as a *cas* gene across all genomes. We simulated a null distribution for each of the 32 families by reassigning the locations of the putative novel *cas* gene in each genome to the location of another randomly chosen open reading frame (ORF) (10^4^ simulations for each gene).

Similarly, we downloaded all completely assembled prokaryotic genomes from GenBank and searched them against the HMMs built as described above. For each genome, we drew a random location on the genome in order to simulate a null distribution of random gene locations with respect to *cas* across the genomes (repeated 100 times for 100 null distributions). We then calculated the minimum distance (in base pairs) from each of the 32 predicted *cas* to any of the previously annotated *cas* genes across all genomes. One putative novel *cas* gene (NOG10439) was not found in any of these genomes using our E value cutoff (0.01/number of genomes).

### Annotation of putative *cas* genes.

We used a number of tools from the HHsuite and hmmer software packages to perform sensitive profile-profile or profile-sequence searches for distant protein homology (19, 29). We first built HMM profiles of our predicted NOGs using hmmer’s hmmbuild command. To detect homology to known *cas* genes, we downloaded all proteins from UniProtKB that included the keywork “CRISPR” in the protein name field. To this set, we added sequences from the alignments of novel *cas* genes found by Shmakov et al. (4) (ftp://ftp.ncbi.nlm.nih.gov/pub/wolf/_suppl/CRISPRicity/NewProfiles.tar.gz). We then used hmmsearch to compare the HMM for each of the predicted *cas* NOGs to this large set of 111,761 amino acid sequences using an E value cutoff of 0.05.

For broader functional prediction, we used HHblits to search for related genes in uniclust30 and HHsearch to search for homologous domains in Pfam (19). For all HHsuite searches, we used a cutoff probability score of 90% in annotating homologs (HHsuite documentation recommends using their probability score rather than E values, which can be unreliable in this context).

Finally, we used TMHMM to predict whether each gene contained in a predicted NOG coded for a transmembrane protein (30) (proteins predicted by TMHMM to have at least 18 amino acids in transmembrane helices).

The putative type was determined by looking at nearby (within two nodes) known *cas* genes in the network, and type determinations were considered to represent sufficient confidence if at least two neighbors had that type.

### Transcription analysis.

We downloaded three transcriptome sequencing (RNA-seq) data sets spanning multiple domains of life in which *cas* genes had been previously observed to be upregulated postinfection, including two from different Sulfolobus islandicus strains (LAL14/1 and REY15A [16, 17]) and one from Thermus thermophilus HB8 (18). We matched our set of putative novel *cas* genes to the genome for each organism (GCF_000364745.1, GCF_000189555.1, and GCF_000091545.1) using hmmscan and the HMM profiles built for each NOG as described above (E value cutoff of 0.01/number of genes in each genome). Because of the diverse set of experimental approaches and analysis pipelines used to quantify expression across the three data sets, we chose to present expression data as analyzed by the original authors, keeping their *P* values and data normalization without modification. We note that these expression data sets were exclusively from thermophilic organisms and may not have provided the best correspondence to the marine samples used for our network-based predictions. Unfortunately, there is a lack of expression data sets demonstrating upregulated CRISPR immunity during viral infection of marine organisms.

Note that the two S. islandicus data sets used different host strains and different viruses and examined different time scales of infection (hours versus days).

The T. thermophilus HB8 *cas* operons all lie on a single plasmid, as do the majority of the CRISPR arrays possessed by this organism. Agari et al. note that knocking out the host gene *crp* decreases the postinfection expression of plasmid-borne *cas* targeting genes but leads to an increase in the level of expression of the plasmid-borne spacer acquisition machinery (18). We included data from both wild-type and Δ*crp* strains in our analysis (Fig. S4 and S5).
