# Supplementary figures and images for: Network-Based Prediction of Novel CRISPR-Associated Genes in Metagenomes
Source: mSystems. 2020 Jan 14;5(1):e00752-19. doi: 10.1128/mSystems.00752-19 (PMC6967390; doi:10.1128/mSystems.00752-19)

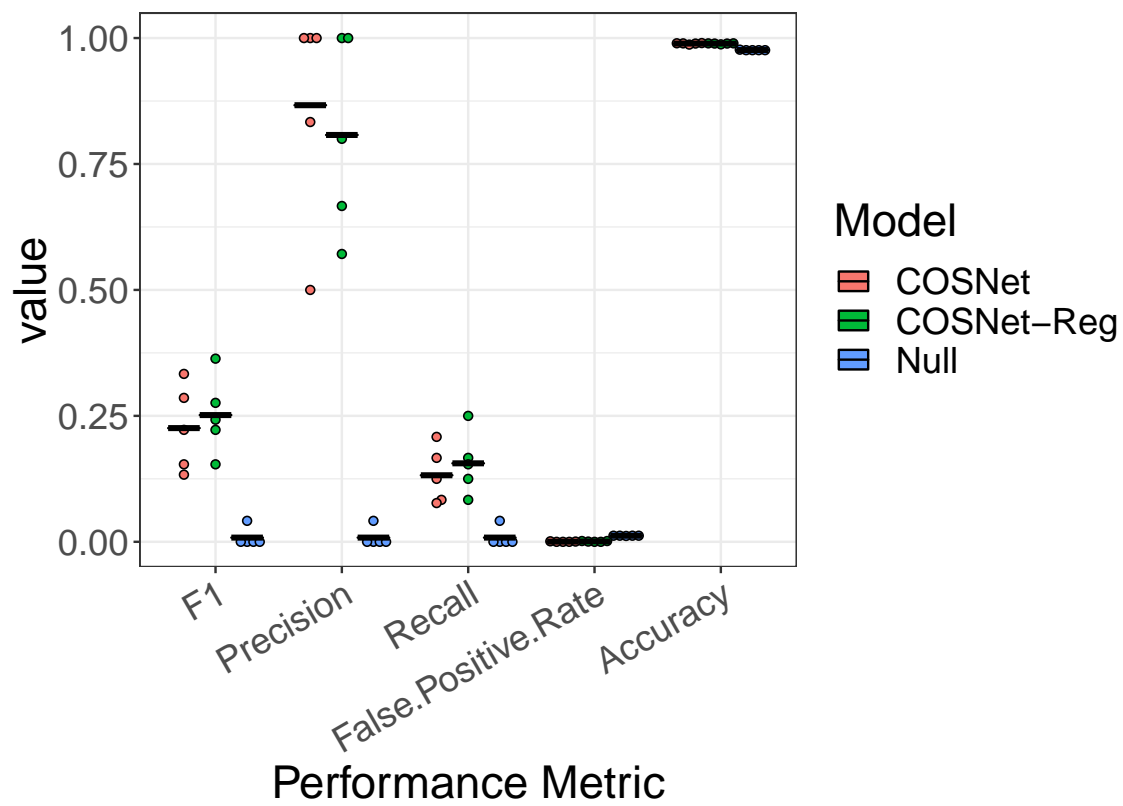

Figure S1

Supplement: FIG S1 [file mSystems.00752-19-sf001.pdf]

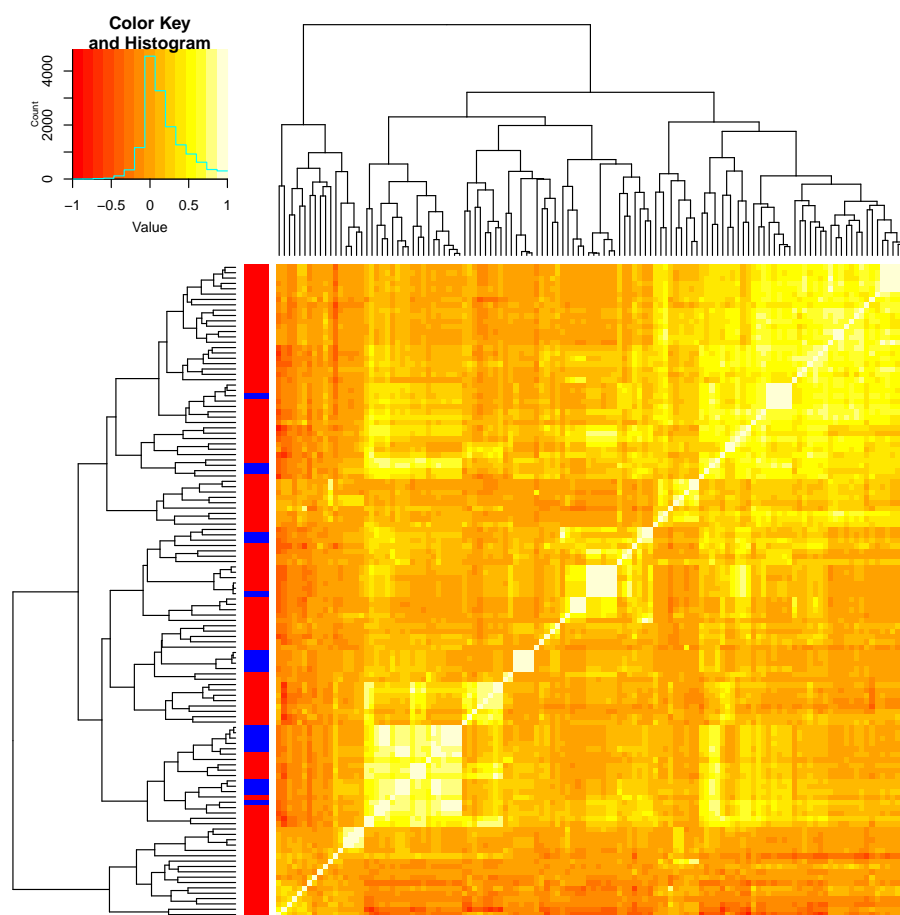

(a)

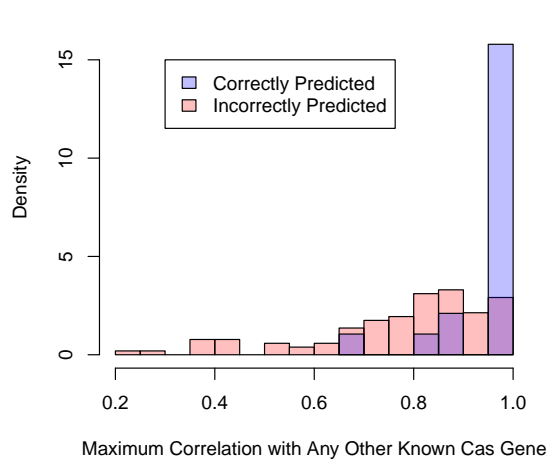

(b)

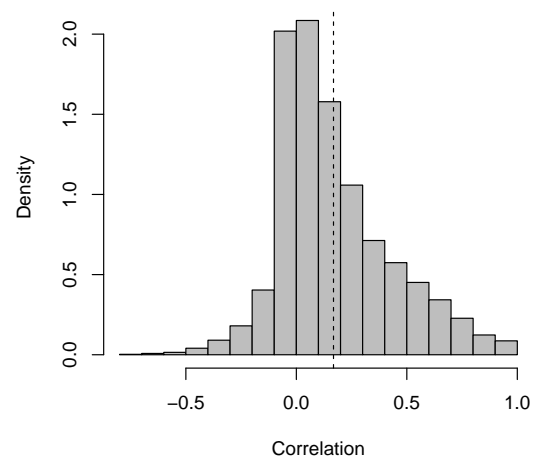

(c)

Figure S2

Supplement: FIG S2 [file mSystems.00752-19-sf002.pdf]

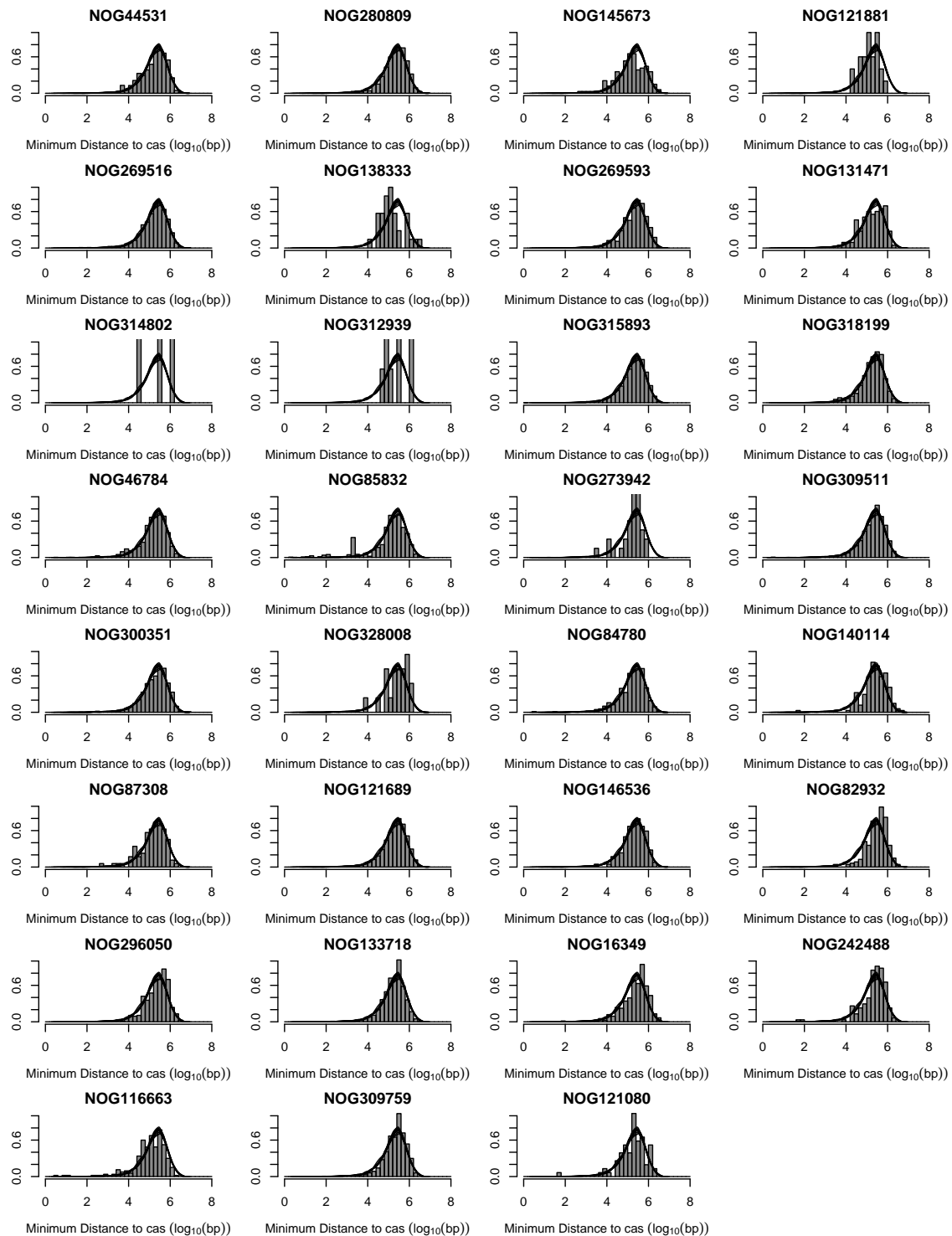

Figure S3

Supplement: FIG S3 [file mSystems.00752-19-sf003.pdf]

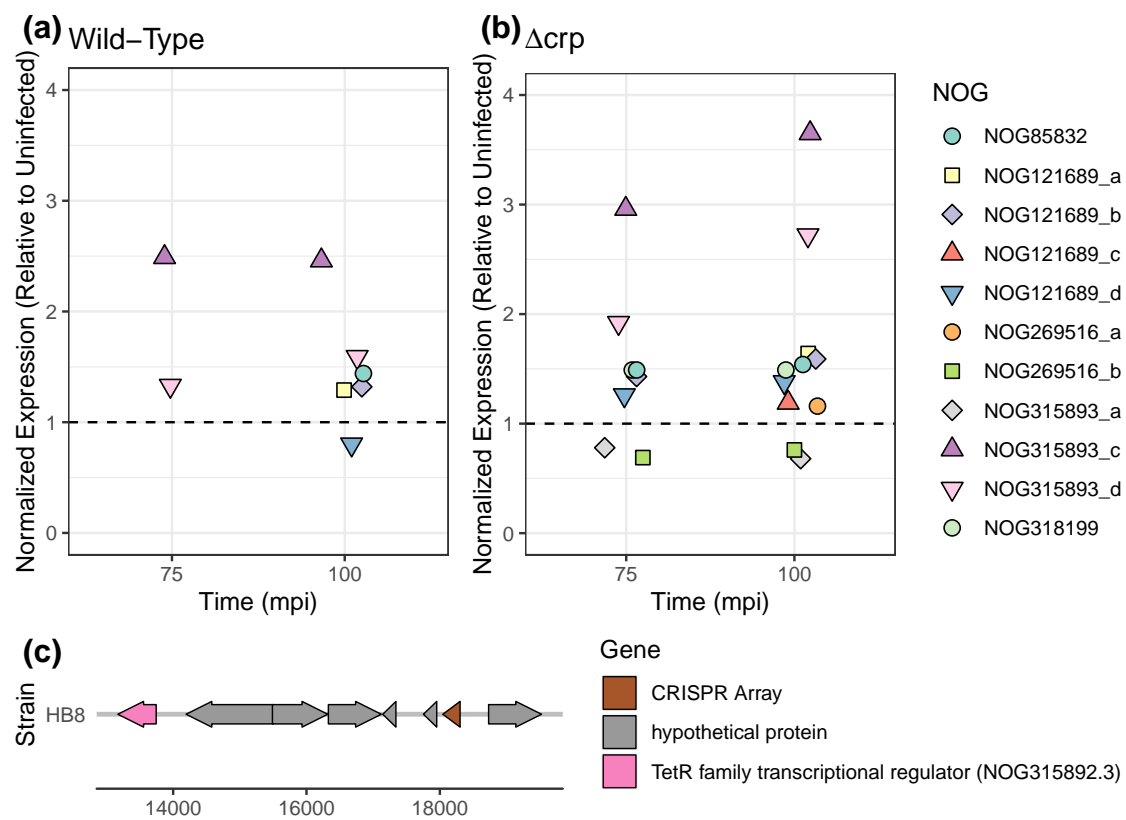

Figure S4

Supplement: FIG S4 [file mSystems.00752-19-sf004.pdf]

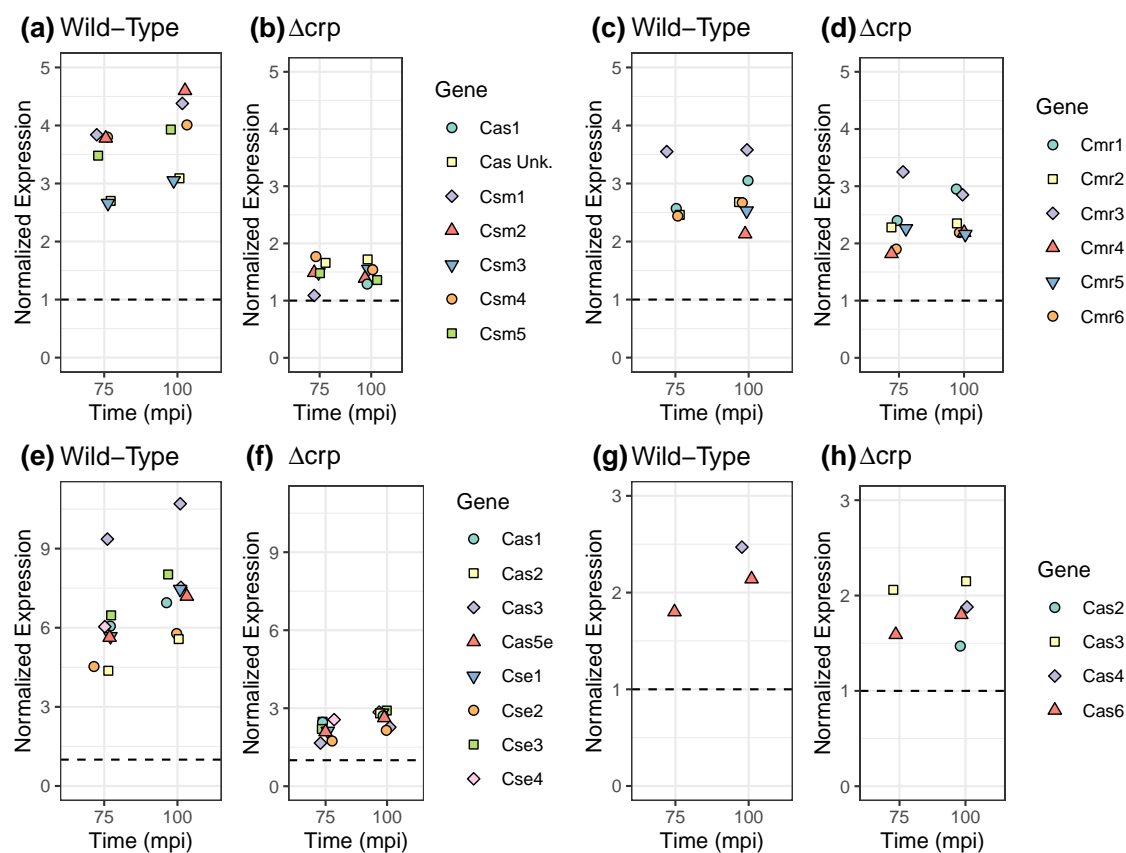

Figure S5

Supplement: FIG S5 [file mSystems.00752-19-sf005.pdf]
