# Supplementary material for: Network-Based Prediction of Novel CRISPR-Associated Genes in Metagenomes
Source: mSystems. 2020 Jan 14;5(1):e00752-19. doi: 10.1128/mSystems.00752-19 (PMC6967390; doi:10.1128/mSystems.00752-19)
Supplement: TABLE S1 [file mSystems.00752-19-st001.pdf]

Table S1:

NOG	E-value	Cas
NOG87308	0.037	CRISPR-associated protein Csx11 OS=Thermococci archaeon OX=2250254 GN=DRN58_06905 PE=4 SV=1
NOG87308	0.042	CRISPR-associated protein Csx11 OS=Candidatus Altiarchaeales archaeon A3 OX=1933927 GN=BWK75_05475 PE=4 SV=1
NOG121080	0.023	CRISPR-associated protein Cas1 OS=Ralstonia solanacearum OX=305 GN=cas1 PE=4 SV=1
NOG145673	0.000039	CRISPR repeat-binding protein OS=Sulfolobus islandicus (strain REY15A) OX=930945 GN=SiRe_1547 PE=4 SV=1
NOG145673	0.000039	CRISPR repeat-binding protein OS=Sulfolobus islandicus (strain HVE10/4) OX=930943 GN=SiH_1625 PE=4 SV=1
NOG145673	0.000046	CRISPR-associated protein OS=Saccharolobus solfataricus OX=2287 GN=SSOP1_0436 PE=4 SV=1
NOG145673	0.000051	CRISPR-associated protein OS=Sulfolobus islandicus OX=43080 GN=DDW12_09115 PE=4 SV=1
NOG145673	0.00017	CRISPR-associated protein OS=Acidianus brierleyi OX=41673 GN=DFR85_08195 PE=4 SV=1
NOG145673	0.00024	CRISPR-associated protein OS=Metallosphaera hakonensis JCM 8857 = DSM 7519 OX=1293036 GN=DFR87_07565 PE=4 SV=1
NOG145673	0.00045	CRISPR-associated protein (Fragment) OS=Candidatus Aramenus sulfurataquae OX=1326980 GN=TQ35_07940 PE=4 SV=1
NOG145673	0.00067	CRISPR-associated protein OS=Acidianus hospitalis OX=563177 GN=DDW13_05870 PE=4 SV=1
NOG145673	0.00075	CRISPR repeat-binding protein OS=Candidatus Aramenus sulfurataquae OX=1326980 GN=ASUL_01385 PE=4 SV=1
NOG145673	0.0011	CRISPR-associated protein OS=Metallosphaera sedula OX=43687 GN=MsedA_2229 PE=4 SV=1
NOG145673	0.0022	CRISPR-associated protein OS=Sulfolobus sp. A20 OX=1891280 GN=BFU36_03535 PE=4 SV=1
NOG145673	0.008	CRISPR repeat-binding protein OS=Acidianus hospitalis (strain W1) OX=933801 GN=Ahos_0975 PE=4 SV=1
NOG145673	0.018	CRISPR-associated protein OS=Acidianus sulfidivorans JP7 OX=619593 GN=DFR86_07725 PE=4 SV=1
NOG145673	0.021	CRISPR-associated protein OS=Acidianus manzaensis OX=282676 GN=B6F84_08715 PE=4 SV=1
NOG145673	0.027	CRISPR-associated protein OS=Candidatus Acidianus copahuensis OX=1160895 GN=CM19_11780 PE=4 SV=1
NOG273942	0.008	Type II CRISPR RNA-guided endonuclease Cas9 OS=Arcobacter defluvii OX=873191 GN=cas9 PE=4 SV=1
NOG273942	0.01	Type II CRISPR RNA-guided endonuclease Cas9 OS=Sulfurimonas sp. SN118 OX=2574727 GN=cas9 PE=4 SV=1
NOG314802	1.9E-19	CRISPR-associated protein OS=Neisseria sp. 10023 OX=1853278 GN=BG910_04095 PE=4 SV=1
NOG314802	2.6E-18	Csm2 family CRISPR-associated protein OS=Neisseria wadsworthii 9715 OX=1030841 GN=csm2 PE=4 SV=1
NOG314802	1.2E-17	CRISPR-associated protein OS=Nitrosomonas mobilis OX=51642 GN=NSMM_150112 PE=4 SV=1
NOG314802	1.5E-16	CRISPR-associated protein OS=Alishewanella aestuarii B11 OX=1197174 GN=AEST_04730 PE=4 SV=1
NOG314802	2E-16	CRISPR-associated protein OS=Alishewanella jeotgali KCTC 22429 OX=1129374 GN=AJE_02506 PE=4 SV=1
NOG314802	4.6E-16	CRISPR-associated protein OS=Thiomicrospira sp. OX=935 GN=DD716_01135 PE=4 SV=1
NOG314802	6.6E-16	CRISPR-associated protein OS=Mesorhizobium sp. LSHC420B00 OX=1287292 GN=X759_28235 PE=4 SV=1
NOG314802	8.1E-16	CRISPR-associated protein OS=Alcanivorax sp. OX=1872427 GN=COB00_09005 PE=4 SV=1
NOG314802	8.4E-16	CRISPR-associated protein OS=Leucothrix sp. IMCC9719 OX=1481894 GN=DKT75_21190 PE=4 SV=1

NOG	E-value	Cas
NOG314802	6.8E-15	CRISPR-associated protein OS=Vibrio parahaemolyticus OX=670 GN=CGI48_23620 PE=4 SV=1
NOG314802	8E-15	CRISPR-associated protein OS=Oceanobacter sp. OX=2030817 GN=COA68_07085 PE=4 SV=1
NOG314802	1.1E-14	CRISPR-associated protein OS=Desulfobulbaceae bacterium OX=2053307 GN=C0613_08670 PE=4 SV=1
NOG314802	2.6E-14	CRISPR-associated protein OS=Rhodothermaceae bacterium OX=2026787 GN=CMM85_10150 PE=4 SV=1
NOG314802	6.4E-14	CRISPR-associated protein OS=Gammaproteobacteria bacterium OX=1913989 GN=COB94_01640 PE=4 SV=1
NOG314802	7.9E-14	CRISPR-associated protein (Fragment) OS=Nitrospiraceae bacterium OX=2026770 GN=DCP24_00595 PE=4 SV=1
NOG314802	9.3E-14	CRISPR-associated protein OS=Thiotrichales bacterium OX=2026796 GN=COB61_01340 PE=4 SV=1
NOG314802	1.1E-13	CRISPR-associated protein OS=Bathymodiolus azoricus thioautotrophic gill symbiont OX=235205 GN=BAZSYMA_ACONTIG00031_5 PE=4 SV=1
NOG314802	1.3E-13	CRISPR-associated protein OS=Colwellia sp. 12G3 OX=2058299 GN=CXF71_10070 PE=4 SV=1
NOG314802	4E-13	CRISPR-associated protein OS=Rodentibacter genomosp. 1 OX=1908264 GN=BKK54_10920 PE=4 SV=1
NOG314802	6E-13	CRISPR-associated protein OS=Proteobacteria bacterium OX=1977087 GN=CSB47_11555 PE=4 SV=1
NOG314802	6.9E-13	CRISPR-associated protein OS=Deltaproteobacteria bacterium HGW-Deltaproteobacteria-19 OX=2013746 GN=CVU61_09135 PE=4 SV=1
NOG314802	1.8E-12	CRISPR-associated protein OS=Bathymodiolus thermophilus thioautotrophic gill symbiont OX=2360 GN=MS2017_2059 PE=4 SV=1
NOG314802	3.8E-12	CRISPR-associated protein Csm2 OS=Anabaena sp. AL09 OX=1710891 GN=AN490_18135 PE=4 SV=1
NOG314802	1.1E-11	CRISPR-associated protein Csm2 OS=Nostoc sp. ATCC 43529 OX=1840705 GN=A6S26_09815 PE=4 SV=1
NOG314802	1.1E-11	CRISPR-associated protein OS=Methylomonas sp. OX=418 GN=CTY19_18695 PE=4 SV=1
NOG314802	1.3E-11	CRISPR-associated protein Csm2 OS=Anabaena sp. WA113 OX=1710889 GN=AN488_11720 PE=4 SV=1
NOG314802	1.9E-11	CRISPR-associated protein Csm2 OS=Anabaena sp. LE011-02 OX=1710892 GN=AN482_16780 PE=4 SV=1
NOG314802	1.9E-11	CRISPR-associated protein Csm2 OS=Aphanizomenon flos-aquae LD13 OX=1710894 GN=AN481_12355 PE=4 SV=1
NOG314802	1.9E-11	CRISPR-associated protein Csm2 OS=Anabaena sp. UBA12330 OX=2055756 GN=DIU28_01505 PE=4 SV=1
NOG314802	4.4E-11	CRISPR-associated protein Csm2 OS=Anabaena sp. WA102 OX=1647413 GN=AA650_11205 PE=4 SV=1
NOG314802	6.3E-11	CRISPR-associated protein OS=Novosphingobium pentaromativorans OX=205844 GN=DI555_08075 PE=4 SV=1
NOG314802	9.7E-11	CRISPR-associated protein OS=Kangiella sp. OX=1920245 GN=COA86_14880 PE=4 SV=1
NOG314802	1.3E-10	CRISPR-associated protein OS=Arcobacter sp. CECT 8985 OX=1935424 GN=CRU93_01440 PE=4 SV=1
NOG314802	3.5E-10	CRISPR-associated protein Csm2 (Fragment) OS=Anabaena sp. MDT14b OX=1710886 GN=AN485_10795 PE=4 SV=1
NOG314802	5.6E-10	CRISPR-associated protein OS=Deltaproteobacteria bacterium CG_4_10_14_3_um_filter_60_8 OX=1973960 GN=COZ12_02020 PE=4 SV=1
NOG314802	0.000000001	Csm2 family CRISPR-associated protein OS=Helicobacter cetorum (strain ATCC BAA-540 / MIT 99-5656) OX=1163745 GN=HCD_02715 PE=4 SV=1
NOG314802	0.000000002	CRISPR-associated protein OS=Candidatus Methyloumidiphilus alinensis OX=2202197 GN=DM484_12170 PE=4 SV=1
